# Do practitioners and friends support patients with coronary heart disease in lifestyle change? a qualitative study

**DOI:** 10.1186/1471-2296-14-126

**Published:** 2013-08-28

**Authors:** Judith A Cole, Susan M Smith, Nigel Hart, Margaret E Cupples

**Affiliations:** 1 UKCRC Centre of Excellence for Public Health, Queen’s University Belfast, Dunluce Health Centre, 1 Dunluce Avenue, Belfast BT9 7HR, Northern Ireland; 2 Department of General Practice, Royal College of Surgeons, Beaux Lane House, Mercer Street, Dublin, 2, Ireland; 3 Centre for Medical Education, Queen’s University Belfast, Dunluce Health Centre, 1 Dunluce Avenue, Belfast BT9 7HR, Northern Ireland

**Keywords:** Coronary heart disease, General practice, Lifestyle change, Secondary prevention, Diet, Physical activity, Barriers, Facilitators

## Abstract

**Background:**

Healthy lifestyles help to prevent coronary heart disease (CHD) but outcomes from secondary prevention interventions which support lifestyle change have been disappointing. This study is a novel, in-depth exploration of patient factors affecting lifestyle behaviour change within an intervention designed to improve secondary prevention for patients with CHD in primary care using personalised tailored support. We aimed to explore patients’ perceptions of factors affecting lifestyle change within a trial of this intervention (the SPHERE Study), using semi-structured, one-to-one interviews, with patients in general practice.

**Methods:**

Interviews (45) were conducted in purposively selected general practices (15) which had participated in the SPHERE Study. Individuals, with CHD, were selected to include those who succeeded in improving physical activity levels and dietary fibre intake and those who did not. We explored motivations, barriers to lifestyle change and information utilised by patients. Data collection and analysis, using a thematic framework and the constant comparative method, were iterative, continuing until data saturation was achieved.

**Results:**

We identified novel barriers to lifestyle change: such disincentives included strong negative influences of social networks, linked to cultural norms which encouraged consumption of ‘delicious’ but unhealthy food and discouraged engagement in physical activity. Findings illustrated how personalised support within an ongoing trusted patient-professional relationship was valued. Previously known barriers and facilitators relating to support, beliefs and information were confirmed.

**Conclusions:**

Intervention development in supporting lifestyle change in secondary prevention needs to more effectively address patients’ difficulties in overcoming negative social influences and maintaining interest in living healthily.

## Background

European guidelines advocate that secondary prevention for coronary heart disease (CHD) should include adopting a healthier diet and increasing physical activity (PA) [1]. Those with responsibility for healthcare should support people in their efforts to change their lifestyle behaviours [2]. Interventions designed to improve secondary prevention improve life expectancy [3], processes of care and clinical outcomes [4]. However, EUROASPIRE surveys show persisting unhealthy lifestyle trends and inadequate adoption of prevention measures within clinical practice [5].

Murray et al. [6] stated that there was a lack of clarity about the main barriers and facilitators to lifestyle change among high risk patients. In their review incorporating 33 qualitative studies they presented five main themes – emotions, beliefs, information and communication, friends and family support, and cost/transport – as major influences of behaviour change. They concluded that further investigation was needed to determine which barriers and facilitators affected participation in behaviour change programmes.

A new framework, the ‘behaviour change wheel’, was designed [7] in order to address the limitations of existing behaviour change frameworks, upon which many lifestyle interventions are based. This highlights a need to consider conditions internal to individuals, in addition to those within their social and physical environment, which need to exist before behaviour change can occur.There remains a gap in knowledge regarding the best approach to helping patients make and maintain lifestyle change [8].

Previously an intervention designed to support patients with CHD in lifestyle change and take into account known barriers to change was developed in the SPHERE Study (Table 1) and evaluated in a randomised controlled trial [9]. Participants were invited to regular four-monthly consultations, during which motivational interviewing techniques were employed to address barriers to change and targets and goals were set and reviewed. Outcome data, collected in 2008, 18 months after the start of the intervention in each general practice (July 2005 to July 2007) showed that, whilst hospital admissions were reduced, no other benefits [10] or effects on lifestyle behaviours were found.

**Table 1:** The SPHERE Study

	
Design	Randomised controlled trial.
Setting	General practices in Northern Ireland and Republic of Ireland.
Participants	903 patients with CHD within 48 practices.
Patient eligibility	Existing CHD (myocardial infarction, coronary artery bypass graft or angioplasty, angina). Patients with significant mental or physical illness excluded.
Intervention	Tailored care plans for practices (training in prescribing and behaviour change, administrative support, quarterly newsletter); tailored care plans for patients (motivational interviewing, goal identification, target setting, review four monthly until 18 month follow up).
Outcome measures	Included: primary: blood pressure, total cholesterol, hospital admissions; secondary: body mass index, exercise (Godin Leisure-Time questionnaire), diet (DINE questionnaire), smoking status.
Results	No significant differences between intervention and control groups relating to blood pressure and cholesterol. Hospital admissions for intervention group significantly decreased compared to control group.

The current study aimed to expand current knowledge of what helps and hinders patients with CHD to make and maintain lifestyle changes, by using a mixed methods approach [11]: qualitative methods were used to explore the experiences of those who participated in the tailored intervention of the SPHERE Study and to help explain measurement data derived from validated questionnaires.

## Methods

Ethical approval for this study was granted by the Office for Research Ethics Committees (Northern Ireland), ref 10/NIR03/11 (April 2010), and the Irish College of General Practitioners Research Ethics Committee (May 2010).

We invited 23 practices, purposively selected to include different sizes and locations, from the original 48 practices which took part in SPHERE (8 in Northern Ireland (NI); 15 in Republic of Ireland (RoI)). Within these practices we selected participants, based on their responses to validated questionnaires at baseline and 18 month follow-up, and invited them to participate in semi-structured interviews. A maximum variation sampling strategy [12] identified individuals with varying baseline values and levels of change in physical activity (PA) (Godin questionnaire [13]) and in dietary fibre (Dietary Instrument for Nutrition Education [14] (DINE)). The DINE questionnaire provides an objective measure of both dietary fibre and fat however we focused on fibre. Participants were asked about both fat and fibre intake during the semi structured interviews.

Maximum variation sampling strategy also identified individuals with different genders, ages, areas of residence, diagnoses (angina/ myocardial infarction) and time since diagnosis, in both intervention and control groups. We included control group patients in order to determine if their views differed from those of patients who received the intervention. The opportunity for exclusion was provided for individuals with significant mental health or physical illness, whose general practitioner considered contact may cause distress, or who would be unable to participate in an interview.

Interviews lasting 20–60 minutes were conducted by the researcher (JC) (December 2010 to September 2011) in participants’ own homes or their local general practice premises. A semi-structured interview schedule was designed (Table 2). The primary questions related to what participants thought of the SPHERE Study, the information booklet they had received, their health-related beliefs*,* their motivations for living a healthy lifestyle and barriers to lifestyle change.

**Table 2:** Semi structured interview schedule

	
**Background and the study**	Overall, what did you think of the Sphere study?
	In terms of the management of your heart disease, was it conducted any differently during the study than before the study began or after it ended?
	What did you think of the visits you made to the study nurse (every 4 months) and the idea of setting goals and targets to change your exercise and diet?
**The Patient Information Booklet**	What did you think of the booklet? In what way did it affect your motivation for change? How often did you refer to it? What did you think of the self-completion sections in the booklet? How useful were these to you? How could the booklet have been improved?
**For those who failed to increase exercise**	How much / what exercise do you currently do?
	*Motivation:*
	At the beginning of this study: when you set a goal to increase the level of exercise you do, how achievable did you think this goal was? What do you think stopped you from achieving that goal?How much do other worries/issues in your life contribute to a lack of motivation to exercise?
	Why did your exercise levels decrease? If you did enjoy exercise in the past, can you remember why?
	How do you think you would feel if you were successful in gradually increasing your exercise?
	*Health beliefs:*
	How did you feel that doing more exercise would affect your health (physical/mental)?
	How do you feel when your doctor tells you that more exercise will be good for your overall health? How much would it mean to you to feel better in your overall health?
**For those who increased level of exercise**	How much / what exercise do you currently do? Before the study, how much exercise did you do? How did you increase your levels of exercise? How did you maintain that level?
	How did you overcome previous barriers to exercising more?
	How does doing more exercise make you feel? (physically, mentally)
**For those who failed to improve fibre intake**	*Motivation:*
	When you set a goal during this study to increase your fibre intake/fruit and vegetables, how achievable did you believe this goal was? When you tried to make the change, what do you think stopped you from achieving it?
	*Health beliefs:*
	When your doctor/nurse explained that improving your diet would benefit your overall health, how motivated did that make you feel?
	When it came to actually trying to achieve your goal, to what extent did you believe it would have real benefits?
**For those who improved fibre intake**	What changes did you make to your diet? When you decided to make changes to your diet, did you believe that you could achieve this and maintain the changes?
	Why do you think you succeeded in making changes? How easy or difficult was it to make the changes, in terms of finding healthier food in the shops and buying it (more expensive, less or no difference)?

Interviews were transcribed and then analysed using a thematic framework [15] and the constant comparative method, facilitated by using NVivo [16]. Data collection and analysis were iterative and continued until data saturation was achieved. For validation purposes and to pursue discovered evidence, themes emerging in earlier interviews were explored further in later interviews. We followed the Consolidated criteria for Reporting Qualitative research (COREQ) principles [17], a detailed outline of which is presented in Additional file 1 and includes consideration of the research team, methods, study setting, results and analyses.

## Results

Of 138 individuals invited to participate in this current study, 84 replied: 64 agreed to take part. Forty-five participants were interviewed (Table 3): 15 expressed willingness to participate but could not be contacted, 4 did not attend interview appointments (one was unwell). One interview was inaudible and was not transcribed.

**Table 3:** Characteristics of invitees and participants to study

	**Invitees**		**Participants**	
	**Intervention**	**Control**	Intervention (%)*	Control (%)*
Gender				
M	47	48	21 (44.7)	17 (35.4)
F	21	22	3 (14.3)	4 (18.2)
Total	68	70	24 (35.3)	21 (30.0)
Age (yrs)				
<60	6	6	2 (33.3)	3 (50.0)
60–70	25	30	10 (40.0)	8 (26.7)
>70	37	34	12 (32.4)	10 (29.4)
Godin				
Baseline				
Inactive (<24)	35	33	12 (34.3)	11 (33.3)
Active (≥24)	13	14	6 (46.2)	4 (28.6)
Score unavailable	20	23	6 (30.0)	6 (26.1)
Increased at follow-up	20	14	7 (35.0)	5 (35.7)
Decreased at follow-up	12	20	6 (50.0)	4 (20.0)
No change at follow-up	7	7	3 (42.9)	3 (42.9)
Score unavailable or N/A	29	29	8 (27.6)	9 (31.0)
Increased at interview			8	6
Decreased at interview			6	3
No change at interview			5	4
Score unavailable or N/A			5	8
DINE				
Baseline				
Low fibre <30	21	17	10 (47.6)	3 (17.6)
Medium 30–40	24	26	8 (33.3)	8 (30.8)
High >40	21	25	6 (28.9)	10 (40.0)
Score unavailable	2	2	0	0
Increased at follow-up	21	21	7 (33.3)	4 (19.0)
Decreased at follow-up	40	39	16 (40.0)	15 (38.5)
No change at follow-up	1	2	1 (100)	1 (50.0)
Score unavailable	6	6	0	1 (16.7)
Increased at interview			11	11
Decreased at interview			12	7
No change at interview			0	1
Score unavailable			1	2

*Percentage represents number of participants relative to number in category invited.

Analysis identified four main themes: facilitators of change, personal beliefs, barriers to change and information. Participants’ comments, supporting the findings, are shown in Tables 4, 5, 6 and 7, and are coded by: Gender (M/F), age (years), region (NI or RoI), Godin (G) change in score from baseline to 18 month follow-up, (current Godin score, at end of interview), DINE (D) change in score from baseline to 18 months, (current DINE score, at end of interview). A positive change in score indicates improved PA or diet (from baseline to 18 month follow-up); a negative change indicates less activity/dietary fibre. Godin scores less than 24 indicate less than recommended levels of PA; DINE scores <30, 30–40 and >40 indicate dietary fibre intake which is low/medium/high, respectively. Scores not available or invalid are indicated by ‘NA’.

**Table 4:** Facilitators of change

	
Professional support	*‘They (the health professionals) know the exercise I’m doing, which was good because sometimes when you do exercise you have no breath. You think ‘I’m going to kill myself”* (F, 81, NI, G+16, D+8).
	*‘I cannot speak highly enough of that practice … such great care, the time they have for you, they get to know you as a person.’* (M, 72, NI, G+24.5 (105), D NA (22)).
	*‘If I come in with a question they’ll go to a lot of trouble to answer.’* (M, 69, RoI, G+37 (76), D-4 (31)).
	*‘I bought a wok as opposed to the frying pan. It was Doctor (XX) who suggested the wok. He said you’ll eat healthier, and I did.’* (M, 56, NI, G0 (0), D+15 (59)).
Setting goals	*‘I had to face (nurse) every four months. I didn’t want to come in and put up the half a stone (target) when I could come in and have lost a few pounds … that was one of the most beneficial things, the target setting.’* (M, 69, RoI, G+37 (76), D-4 (31)).
	*‘You don’t want to disappoint somebody when you’ve entered into some sort of pact … .’* (M, 56, NI, G0 (0), D+15 (59)).
Enjoyment	*‘I’d often be kind of down and you’d go out for a walk, meet someone.... maybe talk.... and when you come back you feel that bit better.’* (M, 65, RoI, G NA (21), D+20 (71)).
	*‘I walk a couple of miles every other day there, up round the mountain.... it certainly helps.’* (M, 65, NI, G+3 (9), D-2 (17)).
	*‘I found I liked gardening, it’s got an objective.’* (M, 69, RoI, G+37 (76), D-4 (31)).
	*‘With fruit, it has its own taste and its own attraction.... you don’t have to add sauce, vinegar, salt or anything else to it. You get into the way of it, it tastes lovely.’* (M, 56, NI, G0 (0), D+15 (59)).
Fears	*‘I walk about 20 miles a week, I don’t want any trouble again.... if I can do anything to keep myself right I’ll do it, whatever the cost. Fear.... fear is a great thing.’* (M, 65, RoI, G NA, (21), D+20 (71)).
	*‘You have to think about your family. What if something happens to you?’* (M, 65, NI, G+3 (9), D-2 (17)).
Social networks	*‘She does the cooking so she has control over what I’m eating. I do have an odd biscuit but instead of a packet a night it’s two or three a year…I would never have thought of walking up and down the house, she said oh that back corridor is 90 feet long, work that out in miles and do enough to walk a mile.’* (M, 69, RoI, G+37 (76), D-4 (31)).
	*‘We would go for a walk in the park, you hear all the gossip (laughs). My friends … coaxing me into going (swimming).’* (F, 74, NI, G-3 (9), D-28 (57))
	*‘It was very useful. Twenty of us met and discussed our problems between us.’* (M, 65, RoI, G NA (21), D+20 (71)).
	*‘Have to be (fit), because the wife’s very ill.’* (M, 74, NI, G NA (41), D+7 (29)).
	*‘I have a very demanding dog, I walk maybe six to eight miles every day. Without her I don’t think I’d be around at all.’* (M, 74, RoI, G+21 (63), D-6 (20)).

**Table 5:** Personal beliefs

	
Personal beliefs	*‘(Exercise) keeps my weight down. The lighter I am the less out of breath I’d be. I’ve started eating more fibre, more fish. I feel far more healthier. My cholesterol has even improved.’* (M, 65, NI, G NA (41), D+6 (29))
	*‘If your thinking doesn’t change you’re not really doing it for the right reasons. You’re either press ganged or somebody’s, you know, coercing or duress.’* (M, 56, NI, G0 (0), D+15 (59))
	*‘I’d be concerned that I’d drop dead if I did too much. At my age I feel there’s nothing to be gained by it.’* (F, 77, RoI, G-6 (0), D-5 (36)).

**Table 6:** Barriers to change

	
Lack of professional support	*‘(GP) never mentions it (diet). Maybe he’s given up.’* (M, 61, RoI, G +2.5 (39), D -9 (26)).
	*‘If you’re going to them you will have to pay for it – my doctor’s 70 euros.’* (M, 54, RoI, G NA (13), D-3 (49)).
Temptations and treats	*‘I was just bored, I’ve fallen into sneaking (sweets).’* (M, 61, NI, G0 (0), D+22 (32))
	*‘I should do these exercises which I find very boring – sometimes I just don’t bother.’* (F, 74, NI, G-3 (9), D-28 (57)).
Unhelpful social contacts	*‘I’ve a grandchild, she’ll come over and we’ll have chips, and then we’ll have chocolate....’* (M, 54, RoI, G NA (13), D-3 (49)).
	*‘I would to get into the early morning swim but the wife isn’t too keen (laughs), on anything with me getting up so early.’* (M, 73, NI, G+18 (3), D-1 (20)).
	*‘Your mates all used to tease you… they just tormented you… laughed at me with fish in the morning for breakfast.’* (M, 74, NI, G NA (0), D-1 (13)).
	*‘If I take one cigarette out of my wife’s packet I’m back again, it’s making it harder for me.’* (M, 61, S, G-22, D-7).
	*‘My daughter, if she’s having a beer or a glass of wine I’ll have a beer. If she wasn’t .... I probably would take less.’* (M, 61, S, G +2.5, D -9).
Personal problems	*‘With my breast removed I don’t like to go swimming.’* (F, 74, NI, G-3 (9), D-28 (57)).
	*‘I would feel embarrassed at not being able to do it myself.’* (M, 56, NI, G0 (0), D+15 (59)).
	*‘I have arthritis.... I can’t go for a walk, the legs hurt.’* (M, 78, RoI, G NA (15), D-10 (75)).
	*‘Because I’m a diabetic, it’s more frightening.... my feet are a problem and my sight’s another thing. The heart would be the least of my troubles.’* (F, 77, RoI, G-6 (0), D-5 (36)).
	*‘We used to go walking almost every day, but the past four months with this building it all fell by the wayside. It’s a nightmare, it’s really destroyed our lifestyle.’* (M, 66, RoI, G+2.5 (39), D-9 (26)).

**Table 7:** Information

	
Valued information	*‘I talked to the doctor and the dietician, I dropped to ten and a half stone, I listened to what she said.’* (M, 74, NI, G NA (41), D+7 (29)).
	*‘I thought the (SPHERE) booklet was a good help… the practice nurse went through it with me… then I went through it periodically when I came across it in the house.’ (M, 65, NI, G NA (41), D+6 (29)).*
Inadequate information	*‘Where do you go when you want to know something? I mean, somebody says you can look it up on the computer.... but where....?’* (F, 77, RoI, G-6 (0), D-5 (36)).
	*‘Some people say white bread’s bad for you, brown bread’s good for you, others say the opposite so who do you believe?’* (M, 65, NI, G NA, D-20 (20)).
	*‘In the brochures from the heart association they had a thing about.... beta blockers were a waste of time.... you never know, you could be taking the wrong tablet.’* (M, 75, NI, G+63 (119), D-3 (60)).
	*‘Some of it can be very difficult to read. They could.... cut away a lot of the medical terms.’* (M, 54, RoI, G NA (13), D-3 (49)).
	*‘Something where you don’t have to get a dictionary out to look up the words.’* (F, 63, RoI, G NA, D+35 (45)).
	*‘They didn’t know.... whether it was the stomach, whether it was the heart.’* (M, 71, NI, G-3 (21), D-1 (28)).
	*‘Some say it was a heart attack and some say it was a stroke.’* (M, 65, NI, G NA, D-20 (20)).
Information not translated into practice	*‘I read the Daily Mail from cover to cover. Tuesday’s Mail is medical health and sometimes it’s very good.’* (F, 86, NI, G NA, D-10 (30)).
	*‘When you go to your doctor you can say I was reading about this on the net and he can explain it to you.’* (M, 54, RoI, G NA (13), D-3 (49)).
	*‘It (SPHERE booklet) sets out everything you need to know… it’s an excellent booklet… I didn’t refer to it that much… I would have had a look through it at the very, very beginning.’ (M, 48, RoI, G-5 (35), D-9 (28)).*

### Facilitators of change

Factors which motivated participants towards healthier lifestyles included (a) professional support, (b) setting goals, (c) enjoyment, (d) fears, and (e) social networks, including pets.

#### (a) Professional support

Participants spoke of the confidence they gained from attending groups such as cardiac rehabilitation which were led by professionals with expert knowledge. Whilst they found benefit in meeting other people with similar experiences, they believed it was important to have support from people who were qualified to provide information and supervise activity. Other comments indicated appreciation of an established relationship between individuals and their primary healthcare providers and that practical advice from such professionals influenced change positively. Evidence of good communication with empathy also encouraged change (See Table 4).

#### (b) Setting goals

Intervention group participants said that setting goals and targets collaboratively with a health professional and agreeing a review date gave them a focus for change. No control group participants were able to comment on experiences of goal setting but one said he would be keen to try this approach.

#### (c) Enjoyment

Enjoyment of a healthier way of life and experiencing its benefits was important. Participants spoke of a positive mental health impact from exercise which had a purpose and of the particular appeal of the taste of fruit and the feeling of satisfaction from eating it.

#### (d) Fears

Fear for personal consequences for their health from failing to make changes was a strong motivating factor for some. Others also reported how fear of premature death or disability and the consequences for their family influenced change, although this was not always reflected in positive change in DINE or Godin scores.

#### (e) Social networks

Encouragement from and positive influence of family and friends were important. Some participants commented on the value of peer support, reflecting on shared experiences. Others revealed how caring responsibilities motivated attention to self-care.

Pets were included in comments about social responsibilities. Dog ownership was a strong motivator for PA, with the over-riding influence appearing to be that of satisfying the dog’s wishes rather than to improve their own health – although that ‘side-effect’ was appreciated.

### Personal beliefs

Participants who reported beliefs that positive changes would benefit their health made these changes: their beliefs were reinforced when they experienced positive results from their efforts. They acknowledged the need for taking personal responsibility for their behaviours.

Several participants who reported beliefs that PA and a healthy diet had no effect, or a harmful effect, on health had not changed their lifestyles, or had reduced their Godin or DINE scores. Some individuals reported fears, not ameliorated by any professional guidance, that exercise could do harm (Table 5).

### Barriers to change

These included (a) lack of professional support, (b) temptations and treats (c) unhelpful social contacts and (d) personal problems (Table 6).

#### (a) Lack of professional support

Some participants denied any recollection of receiving lifestyle advice from a health professional. Others suggested, with apparent disappointment, that their failure to respond to advice led to GPs ceasing to offer it. Several in the RoI reported that they received little professional support but others also reported that they often failed to seek support from their GP because of the cost of each consultation (primary health care in the RoI is only free (through the General Medical Services scheme) to those deemed less able to pay).

#### (b) Temptations and treats

The associations of unhealthy food, including chocolates and ‘treats’, with pleasure were insurmountable temptations for some individuals, especially when combined with social pressures such as being a guest or receiving gifts. Similarly but by contrast, healthy foodstuffs lacked appeal. Boredom was blamed for lapses in healthy habits, emphasising a need for diversity in behaviour change planning.

#### (c) Unhelpful social contacts

While many participants spoke of family or friends supporting change, others reported not just a lack of support discouraging change but strong negative influences, from cultural ‘norms’ (viewing chips and chocolates as ‘treats’). Within their social networks many participants encountered people who lacked understanding of the relevance of unhealthy eating habits and lack of physical activity for health and who appeared to be unaware of the difficulty of the challenge of lifestyle change for individuals with CHD.

Whilst the focus of our work was on diet and physical activity, participants’ comments included reference to smoking and alcohol and reflected how their everyday living environment and social contacts could have a wider negative impact on their adoption of healthy lifestyle habits.

#### (d) Personal problems

In relation to increasing PA some interviewees were reticent to expose their vulnerability suggesting problems of self-esteem. Comorbidities, most commonly arthritis, were cited as barriers to lifestyle change. Several participants who had diabetes reported being more concerned about managing physical problems than engaging in lifestyle change for secondary prevention. Inability to cope with unexpected disruption of daily routines, including prolonged house renovations, upset plans for lifestyle change.

### Information

Some information which participants had obtained about lifestyle and health was reported to be (a) valued, some (b) inadequate and some (c) not translated into practice. (Table 7)

#### (a) Valued information

Participants perceived value in receiving verbal information and literature produced by professionals. Clear advice which was understood encouraged change – this is another aspect of effective professional support. Some participants from intervention practices valued the SPHERE information booklet as a valuable and handy resource to pick up and read whenever they wished.

Information in the popular media also contributed to positive change in diet and was appreciated by participants who reflected on what they perceived as better dissemination of healthy lifestyle messages currently, compared to previous decades.

#### (b) Inadequate information

Some participants said they did not know where to obtain useful information; others commented that they had received inconsistent and confusing information. These perceptions and experiences influenced lifestyle change negatively.

Several participants who said they had not been given a definitive diagnosis of an acute coronary event questioned whether pursuing a healthier lifestyle was actually relevant to them.

#### (c) Information not translated into practice

Despite digesting information on a daily basis, some participants remained physically inactive and did not improve their diet. People said they enjoyed reading about health but this did not translate into behaviour change. Some participants from intervention practices whose Godin and/or DINE scores decreased admitted they had only looked at the SPHERE booklet at the beginning of the study and not since. One avid reader, whose DINE score decreased, admitted she had a ‘sweet tooth’ and was aware of healthy diet advice but because she enjoyed good health and an active lifestyle she believed the advice was not relevant to her.

## Discussion and conclusions

### Summary of main findings

This study presents a unique report of influences relating to lifestyle change, set in the context of a study of an intervention designed to help patients to overcome barriers to changing their PA and diet. The findings highlight the difficulties of following healthy lifestyles in the context of unhelpful social influences, boredom and disrupted daily routines, the impact of personal experience on behaviour and the value patients with CHD derive from personalised support from health professionals in making and maintaining lifestyle changes. Novel information about disincentives and how friends and family can be unhelpful, by encouraging indulgence in unhealthy foods or by discouraging physical activity, is reported.

Many participants whose lifestyle scores decreased lacked experience of satisfactory personalised professional support. They had little faith in professionals and were sceptical that healthy lifestyle advice could help them. Some desired a ‘quick fix’ solution to their health problems and became discouraged when they did not find one. Others described being bored with new exercise or diet regimes and hankered after their former habits – which offered greater attraction. Their comments suggest that they were not convinced of the importance of healthy lifestyles. Some participants reported that their GP never mentioned lifestyle to them: this may indicate that GPs abandon unsuccessful efforts to promote change or that they lack confidence in discussing lifestyle issues [18]. Participants who did not improve found healthy lifestyles more of a daily struggle and appeared to have poor self-motivation and a magnified view of other problems in their lives.

Many who improved their scores had confidence in their own ability to maintain a healthy lifestyle and perceived that social support was less important than professional support. However, social responsibilities were reported as having significant influence: individuals perceived that they needed to be healthy to care for others.

Continuity of relationships in general practice, with trusted health professionals, encouraged patients to adopt advice and maintain healthy lifestyles. Putting advice into practice and having personal experience of its benefits, such as weight loss, reduced cholesterol, improved mood or pleasure (e.g. enjoying the taste of fruit), encouraged change. Those who had improved lifestyle scores had more spontaneous praise for their health professionals than those who did not. They spoke of their trust in the advice they were given and appreciation of personal care, whilst acknowledging that it is their responsibility to make changes in their own lives. Our findings suggest there is value in professionals reviewing their patients’ lifestyle habits regularly. Setting goals for change in collaboration with a health professional was identified consistently as an important motivator by patients who experienced this within the SPHERE Study intervention.

### Strengths and limitations

Our study involved a large sample of participants with a wide age range and location. A semi structured one-to-one interview approach was a favourable way to yield the information which this study sought. However, some questions required individuals to reflect on experiences of approximately three years previously (their experiences of the SPHERE Study had ended in 2008): time lapses may have caused omission of some details.

A further strength of our study is that experiences of change relate to a specific time period. In their review of qualitative literature relating to barriers and facilitators to lifestyle change Murray et al. [6] commented that many papers did not link experiences to any time period, thus limiting comparison of factors affecting change. Also, we examined PA and diet which was planned and undertaken by patients themselves to fit into their daily lives, rather than within a restricted setting or time-limited programme [6]: our findings thus have relevance for long-term lifestyle change.

Our sample included more men than women, although we had intended to include similar numbers of each. Condon & McCarthy [19] also interviewed a predominately male sample. It may be that women participate in such studies less readily because of family responsibilities, or are less interested in discussing their health and lifestyle with a researcher with whom they have not already established a clinical relationship. Overall, patients spoke more about incentives than barriers which, for those who did not improve their PA or diet, suggested a level of denial or unwillingness to report failure, reflecting a degree of reflexivity with the researcher and a potential wish to portray a good impression. Our findings may therefore reflect a bias although data saturation was achieved.

As the interviewer knew patients’ lifestyle scores before meeting them for interview there was potential for her demeanour to be different with those who had succeeded in improving scores compared to those who had failed. However, she does not believe that this was the case as the potential for this bias was recognised a priori and every effort was made to appear objective both when talking to patients on the telephone to arrange an interview and during the interviews themselves.

### Comparison with existing literature

Whilst our finding that patients regarded health professionals as a valued source of support concurs with research [20] which explored patients’ experiences of presenting health information from the internet to their GPs, it contrasts with the suggestion [19] that people regard health professionals as a source of information rather than of support. In the RoI, where people above a certain income threshold pay to visit their GP, participants’ comments suggested that they tended to visit less often and were more likely to use other sources of information such as the internet; however, they derived confidence from their GP, whom they felt understood them, and they valued deeply their support. Indeed, they reported seeking their GP’s endorsement of information obtained from other sources. Nolan & Smith [21] found that GP utilisation was significantly more likely where the service was free. Thus, different systems of healthcare provision may themselves influence the process of lifestyle change.

We report how the influence of enjoying a lifestyle behaviour helps to maintain it, and the positive impact of useful information. Bergman et al. [22] reported the pleasure patients derived from cycling and the feeling of wellness they experienced from walking. Alm-Roijern et al. [23] reported that better knowledge improved adherence to lifestyle changes. Adding to the published evidence, our study found that lifestyles were influenced positively when patients believed the information they received and personal experiences reinforced belief in its value. However, there is often less recognition that unhealthy behaviours are also enjoyable and this research confirms that the pleasure of eating ‘unhealthy’ foods can be hard to resist, particularly in some social contexts. It may lead to more productive dialogue regarding behaviour change if this is explicitly acknowledged.

Positive support from family, friends and peers can facilitate lifestyle change but overprotection can be a barrier to change [24,25]. However, in contrast to previous reports [18,26] of the positive value of social networks in supporting healthy behaviour, our findings emphasise the level of difficulty posed for individuals by social situations where they struggle to make or maintain change. It would appear that this difficulty is exacerbated for individuals with CHD and has not been fully acknowledged, explored or discussed with patients in practice.

Previous studies have also found that goal setting, action plans and structured programmes of care are effective in promoting lifestyle change [27-30] but goal setting itself is not sufficient to effect change [31]. However, behaviour change interventions which are based on psychological theory have more positive results than those delivered by teaching and merely giving information [32]. A collaborative approach involving both professional and patient is necessary, with recognition of the patient’s social networks, responsibilities and culture to help them overcome hindrances to lifestyle change.

### Implications

There is potential to make a positive impact on individuals’ lives by professionals counselling those who are afraid of worsening their health by exercising more or altering their diet, those who feel lifestyle has no effect on their health and those who want to change but don’t know how. Patients need clear, consistent information. Regular review is associated with improved uptake of secondary prevention [29,33]. Regular monitoring of individuals’ lifestyles may give professionals opportunities to identify difficulties patients are experiencing and facilitate lifestyle change. Health professionals may help patients to avoid boredom by discussing new approaches to exercise or healthy eating, to manage disruption to daily routines, to counteract potential negative influences of friends and to exploit social networks for their own benefit. We understand that there are problems for patients putting theory into practice. However, health professionals should be more cognisant of how ready patients are to make lifestyle changes.

Although some individuals believed that their CHD was inherited and were terrified of another event, they perceived that premature death was inevitable and saw no point in making any lifestyle change. We also identified an attitude pervading among some older participants, that it was too late in life to make effective change. Our findings indicate that the current provision of information is inadequate to allow everyone to make fully informed decisions about lifestyle change. Comments indicate value in multi-source provision but there is a need to ensure that the information is consistent in its message and understood by its target audience.

Future research should test new strategies to promote lifestyle change and overcome negative influences, focusing on providing clear information for individuals. Theoretical frameworks which consider factors within individuals and their external environments should continue to be developed and used in intervention designs to promote healthy behaviour. Prospective mixed methods approaches could be adopted to quantify behaviour changes actually made by individuals in response to these interventions, while collecting qualitative data on the lived experiences of those attempting to make these changes in real life settings.

## Consent

Written informed consent was obtained from all participating patients for the publication of this report.

## Competing interests

The authors declare that they have no competing interests.

## Authors’ contributions

JC analysed lifestyle scores for participant selection, contacted practices and recruited participants, carried out the interviews and analysis and drafted the manuscript. SS was involved in the conception of the study and has been involved in writing the manuscript and revising successive drafts. NH shared in the data-analysis and contributed to the writing of the manuscript, revising successive drafts. MC conceived the study, participated in planning the fieldwork and supervised the data collection, shared in the analysis and writing of the manuscript, revising successive drafts. All authors read and approved the final manuscript.

## Pre-publication history

The pre-publication history for this paper can be accessed here:

http://www.biomedcentral.com/1471-2296/14/126/prepub

## Supplementary Material

Additional file 1 Consolidated criteria for reporting qualitative research (COREQ): a 32-item checklist for interviews and focus groups. Click here for file

## References

[B1] De Backer G Ambrosioni E Borch-Johnsen K Brotons C Cifkova R Dallongeville J Ebrahim S Faergeman O Graham I Mancia G Cats VM Orth-Gomer K Perk J Pyorala K Rodicio JL Sans S Sansoy V Sechtem U Sibler S Thomsen T Wood D European guidelines on cardiovascular disease prevention in clinical practice Eur Heart J 2003 24 1601 1610 10.1016/S0195-668X(03)00347-6 12964575

[B2] Cooper A Skinner J Nherera L Feder G Ritchie G Kathoria M Turnbull N Shaw G MacDermott K Minhas R Packham C Squires H Thomson D Timmis A Walsh J Williams H White A Clinical guidelines and evidence review for post myocardial infarction: secondary prevention in primary and secondary care for patients following a myocardial infarction 2007 London: National Collaborating Centre for Primary Care and Royal College of General Practitioners 21290635

[B3] Jolliffe J Rees K Taylor RRS Thompson DR Oldridge N Ebrahim S Exercise-based rehabilitation for coronary heart disease (Review) Cochrane Database Syst Rev 2001 1 CD001800 11279730 10.1002/14651858.CD001800

[B4] Clark AM Hartling L Vandermeer B McAlister FA Meta-analysis: secondary prevention programs for patients with coronary artery disease Ann Intern Med 2005 143 659 72 10.7326/0003-4819-143-9-200511010-00010 16263889

[B5] Kotseva K Wood D De Backer G De Bacquer D Pyorala K Keil U Cardiovascular prevention guidelines in daily practice: a comparison of EUROASPIRE I, II, and III surveys in eight European countries Lancet 2009 373 9667 929 940 10.1016/S0140-6736(09)60330-5 19286092

[B6] Murray J Honey S Hill K Craigs C House A Individual influences on lifestyle change to reduce vascular risk: a qualitative literature review Br J Gen Pract 2012 10.3399/bjgp12X649089 PMC3361119 22687232

[B7] Michie S Van Stralen MM West R The behaviour change wheel: a new method for characterising and designing behaviour change interventions Implement Sci 2011 6 42 10.1186/1748-5908-6-42 PMC3096582 21513547

[B8] Wofford TS Greenlund KJ Croft JB Labarthe DR Diet and physical activity of US adults with heart disease following preventive advice Prev Med 2007 45 295 301 10.1016/j.ypmed.2007.06.013 17643478

[B9] Murphy AM Cupples ME Smith SM Byrne M Leathem C Byrne MC The SPHERE Study. Secondary prevention of heart disease in general practice: protocol of a randomised controlled trial of tailored practice and patient care plans with parallel qualitative, economic and policy analyses Curr Control Trials Cardiovasc Med 2005 6 11 10.1186/1468-6708-6-11 16053525 PMC1208929

[B10] Murphy AW Cupples ME Smith SM Byrne M Byrne MC Newell J Effect of tailored practice and patient care plans on secondary prevention of heart disease in general practice: cluster randomised controlled trial Br Med J 2009 339 b4220 10.1136/bmj.b4220 19875426 PMC2770592

[B11] Creswell J Mixed methods procedures Research design: qualitative, quantitative and mixed methods approaches 2008 3 California: Sage Publications Ltd 203 225

[B12] Marshall MN Sampling for qualitative research Fam Pract 1996 13 522 525 10.1093/fampra/13.6.522 9023528

[B13] Godin G Shephard RJ A simple method to assess exercise behaviour in the community Can J Appl Sport Sci 1985 10 3 141 6 4053261

[B14] Roe L Strong C Whiteside C Neil A Mant D Dietary intervention in primary care: validity of the DINE method for diet assessment Fam Pract 1994 11 375 381 10.1093/fampra/11.4.375 7895964

[B15] Green G Thorogood N Analysing qualitative data Qualitative methods for health research 2009 2 London: Sage Publications Ltd 173 200

[B16] NVivo qualitative data analysis software Version 9 2010 QSR International Pty Ltd

[B17] Tong A Sainsbury P Craig J Consolidated criteria for reporting qualitative research (COREQ): a 32-item checklist for interviews and focus groups Int J Qual Health Care 2007 19 6 349 357 10.1093/intqhc/mzm042 17872937

[B18] Moore S Daniel M Paquet C Dube L Gauvin L Association of individual network social capital with abdominal adiposity, overweight and obesity J Public Health 2009 31 1 175 183 10.1093/pubmed/fdn104 PMC5167564 19153095

[B19] Condon C McCarthy G Lifestyle changes following acute myocardial infarction: patients’ perspectives Eur J Cardiovasc Nurs 2006 5 37 44 10.1016/j.ejcnurse.2005.06.005 16055382

[B20] Bowes P Stevenson F Ahluwalia S Murray E ‘I need her to be a doctor’: patients’ experiences of presenting health information from the internet in GP consultations Br J Gen Pract 2012 10.3399/bjgp12X658250 PMC3481513 23211176

[B21] Nolan A Smith S The effect of differential eligibility for free GP services on GP utilisation in Ireland Soc Sci Med 2012 74 10 1644 1651 10.1016/j.socscimed.2012.02.007 22459189

[B22] Bergman E Bertero C You can do it if you set your mind to it: a qualitative study of patients with coronary artery disease J Adv Nurs 2001 36 6 733 741 10.1046/j.1365-2648.2001.02040.x 11903703

[B23] Alm-Roijern C Stagmo M Udén G Erhardt L Better knowledge improves adherence to lifestyle changes and medication among patients with coronary heart disease Eur J Cardiovasc Nurs 2004 3 321 330 10.1016/j.ejcnurse.2004.05.002 15572021

[B24] Roebuck A Furze G Thompson DR Health related quality of life after myocardial infarction: an interview study J Adv Nurs 2001 34 6 787 794 10.1046/j.1365-2648.2001.01809.x 11422549

[B25] Goldsmith DJ Lindholm KA Bute JJ Dilemmas of talking about lifestyle changes among couples coping with a cardiac event Soc Sci Med 2006 63 2079 2090 10.1016/j.socscimed.2006.05.005 16790307

[B26] Legh-Jones H Moore S Network social capital, social participation, and physical inactivity in an urban adult population Soc Sci Med 2012 74 9 1362 1367 10.1016/j.socscimed.2012.01.005 22410270

[B27] Cupples ME McKnight A Five year follow up of patients at high cardiovascular risk who took part in randomised controlled trial of health promotion Br Med J 1999 319 687 688 10480826 10.1136/bmj.319.7211.687 PMC28222

[B28] Lewin RJP Furze G Robinson J Griffith K Wiseman S Pye M Boyle R A randomised controlled trial of a self-management plan for patients with newly diagnosed angina Br J Gen Pract 2002 52 476 194 201 12030661 PMC1314238

[B29] Murchie P Campbell NC Ritchie LD Simpson JA Thain J Secondary prevention clinics for coronary heart disease: four year follow up of a randomised controlled trial in primary care Br Med J 2003 326 7380 84 87 10.1136/bmj.326.7380.84 12521974 PMC139939

[B30] Handley M MacGregor K Schillinger D Sharifi C Wong S Bodenheimer T Using action plans to help primary care patients adopt healthy behaviors: a descriptive study J Am Board Fam Med 2006 19 224 231 10.3122/jabfm.19.3.224 16672675

[B31] Jackson C Lawton R Knapp P Raynor DK Conner M Lowe C Closs SJ Beyond intention: do specific plans increase health behaviours in patients in primary care? a study of fruit and vegetable consumption Soc Sci Med 2005 60 2383 2391 10.1016/j.socscimed.2004.10.014 15748685

[B32] Hardeman W Griffin S Johnston M Kinmonth AL Wareham N Interventions to prevent weight gain: a systematic review of psychological models and behaviour change methods Int J Obes 2000 24 131 143 10.1038/sj.ijo.0801100 10702762

[B33] Cupples ME McKnight A Randomised controlled trial of health promotion in general practice for patients at high cardiovascular risk Br Med J 1994 309 993 996 10.1136/bmj.309.6960.993 7950723 PMC2541257

