# Supplementary material for: Do practitioners and friends support patients with coronary heart disease in lifestyle change? a qualitative study
Source: BMC Fam Pract. 2013 Aug 28;14:126. doi: 10.1186/1471-2296-14-126 (PMC3765931; doi:10.1186/1471-2296-14-126)
Supplement: Additional file 1 — Consolidated criteria for reporting qualitative research (COREQ): a 32-item checklist for interviews and focus groups. [file 1471-2296-14-126-S1.doc]

**Additional file 1**

**Consolidated criteria for reporting qualitative research (COREQ): a 32-item checklist for interviews and focus groups**

Article: Do practitioners and friends support patients with coronary heart disease in lifestyle change? A qualitative study

Authors: Dr Judith A Cole, Professor Susan M Smith, Dr Nigel Hart, Professor Margaret E Cupples

**Domain 1: Research team and reflexivity**

**Personal characteristics**

1. **Interviewer**

***Which author/s conducted the interviews?***

Judith Cole: this author had no prior knowledge of the participants nor had she any prior involvement in the SPHERE Study.

1. **Credentials**

***What were the authors’ credentials?***

First author BMedSc, MSc; second author MD, MRCPI, MRCGP; third author MD, MRCGP; fourth author MD, FRCGP.

1. **Occupation**

First author PhD student; second, third and fourth authors all academic GPs.

**4 Gender**

***Was the researcher male or female?***

First, second and fourth authors female, third author male.

**5 Experience and training**

***What experience or training did the researchers have?***

The first author had done training in qualitative research techniques and analyses methods, attending several courses and workshops. Second, third and fourth authors all have had training and experience in conducting previous studies related to cardiovascular disease and its management in primary care.

**6 Relationship established**

***Was a relationship established prior to study commencement?***

This study was a follow-up to a larger interventional study which had finished three to four years previously. Patients had had contact with other researchers involved in the original study but the first author was not involved in this so had no prior relationship with participants.

**7 Participant knowledge of the interviewer**

***What did participants know about the researcher?***

The background to the research and that it was being done as part of the researcher’s PhD project was explained to participants in a study information sheet which they received along with their invitation to take part in the study. They knew that she was based at Queen’s University, Belfast.

**8 Interviewer characteristics**

***What characteristics were reported about the interviewer?***

The potential for bias was reported and efforts to minimise this including the interviewer’s objectivity during telephone and face-to-face contact.

**Domain 2: study design**

**9 Methodological orientation**

***What methodological orientation was stated to underpin the study?***

Thematic framework and constant comparative method.

**10 Sampling**

***How were participants selected?***

Practices were selected purposively. Participants were selected using a maximum variation sampling strategy.

**11 Method of approach**

***How were participants approached?***

Invitations were sent from their general practitioner, by mail. If they agreed to participate, by returning their response by mail on a reply slip, the researcher telephoned them to arrange a time for face to face interview.

**12 Sample size**

***How many participants were in the study?***

45; the audio-recording of one participant’s interview was not audible and could not be used in analysis.

**13 Non-participation?**

***How many people refused to participate or dropped out?***

138 were invited, 84 replied to the invitation, 64 agreed to participate, 20 refused (reasons were not sought). 45 were interviewed, 19 were not for various reasons including illness on the day of interview, emergency situations and did not turn up at the agreed time.

**14 Setting**

***Where was the data collected?***

Patients were asked to choose to meet the researcher either their general practice or home; interviews were done in both settings.

**15 Presence of non-participants**

***Was anyone else present besides the participants and researchers?***

In 3 interviews a family member of the participant was present. This had a beneficial effect of prompting participants to remember events and dates, and details relating to their diet or physical activity.

**16 Description of sample**

***What are the important characteristics of the sample?***

Demographic data (age <60 years 11.1%, 60-70 years 40.0%, >70 years 48.9%; gender 84.4% male, 15.6% female)and medical history (all have history of coronary heart disease).

**17 Interview guide**

***Were questions, prompts, guides provided by the authors? Was it pilot tested?***

Yes, the interview approach and semi structured interview schedule design is described in the methods section. Pilot testing of the schedule, including questions, prompts and guides was conducted with 2 subjects prior to the study.

**18 Repeat interviews**

***Were repeat interviews carried out?***

No.

**19 Audio/visual recording**

***Did the research use audio or visual recording to collect the data?***

Data were audio recorded.

**20 Field notes**

***Were field notes made during and/or after the interviews?***

Yes.

**21 Duration**

***What was the duration of the interviews?***

Variable: ranging from 15 to 60 minutes.

**22 Data saturation**

***Was data saturation discussed?***

Yes.

**23 Transcripts returned**

***Were transcripts returned to participants for comment and/or correction?***

No.

**Domain 3: analysis and findings**

**24 Number of data coders**

***How many data coders coded the data?***

Two.

**25 Description of the coding tree**

***Did authors provide a description of the coding tree?***

No.

**26 Derivation of themes**

***Were themes identified in advance or derived from the data?***

Two ‘parent’ themes of barriers and facilitators were identified in advance because this was the aim of the study. Sub themes were derived from the data.

**27 Software**

***What software was used to manage the data?***

NVivo.

**28 Feedback**

***Did participants provide feedback on the findings?***

No.

**29 Quotations presented**

***Were participant quotations presented to illustrate the findings?***

Yes.

***Was each quotation identified?***

They were not identified to preserve anonymity. However, we have coded each quotation by: Gender (M/F), age (years), region (NI or RoI), Godin (G) change in score from baseline to 18 month follow-up, (current Godin score, at end of interview), DINE (D) change in score from baseline to 18 months, (current DINE score, at end of interview).

**30 Data and findings consistent**

***Was there consistency between the data presented and the findings?***

Yes.

**31 Clarity of major themes**

***Were major themes clearly presented in the findings?***

Yes.

**32 Clarity of minor themes**

***Is there a description of diverse cases or discussion of minor themes?***

Yes.
